# What Savages Think of Twins

**Published:** 1875-10

**Authors:** 


What Savages think of Twins.—In Africa, according to Dr. Robert Brown ("Races of Mankind") the birth of twins is commonly regarded as an evil omen. No one, except the twins themselves and their nearest relatives, is allowed to enter the hut in which they first saw the light. The children are not allowed to play with other children, and even the utensils of the hut are not permitted to be used by any one else. The mother is not allowed to talk to any one not belonging to her own family. If the children both live till the end of the sixth year, it is supposed that Nature has accommodated herself to their existence, and they are thenceforth to associate with their fellows. Nor is abomination of twin births restricted to Africa. In the island of Bali, near Java, a woman who is so unfortunate as to bear twins is obliged, along with her husband, to live for a month at the sea-shore or among the tombs, until she is purified. The Khasias of Hindostan consider that to have twins assimilates the mother to the lower animals, and one is frequently put to death. An exactly similar belief prevails among some of the native tribes of Vancouver Island. Among the Ainos, one of the twins is always killed, and in Arebo, in Guinea, both the twins and the mother are put to death.—*From Popular Science Monthly for Sept.*

